# Internalization of transferrin-tagged *Myxococcus xanthus* encapsulins into mesenchymal stem cells

**DOI:** 10.3389/ebm.2024.10055

**Published:** 2024-05-07

**Authors:** Anna N. Gabashvili, Natalya A. Alexandrushkina, Elizaveta N. Mochalova, Daria V. Goliusova, Ekaterina N. Sapozhnikova, Pavel I. Makarevich, Petr I. Nikitin

**Affiliations:** ^1^ Prokhorov General Physics Institute of the Russian Academy of Sciences, Moscow, Russia; ^2^ Institute for Regenerative Medicine, Medical Research and Education Center, Lomonosov Moscow State University, Moscow, Russia; ^3^ Faculty of Medicine, Lomonosov Moscow State University, Moscow, Russia; ^4^ Moscow Center for Advanced Studies, Moscow, Russia; ^5^ Nanobiomedicine Division, Sirius University of Science and Technology, Sirius, Russia; ^6^ Koltzov Institute of Developmental Biology of the Russian Academy of Sciences, Moscow, Russia; ^7^ Laboratory of Cell Biology, Lopukhin Federal Research and Clinical Center of Physical-Chemical Medicine of FMBA, Moscow, Russia

**Keywords:** encapsulins, fluorescence, photoactivatable label, imaging flow cytometry, targeted delivery nanosystem

## Abstract

Currently, various functionalized nanocarrier systems are extensively studied for targeted delivery of drugs, peptides, and nucleic acids. Joining the approaches of genetic and chemical engineering may produce novel carriers for precise targeting different cellular proteins, which is important for both therapy and diagnosis of various pathologies. Here we present the novel nanocontainers based on vectorized genetically encoded *Myxococcus xanthus* (Mx) encapsulin, confining a fluorescent photoactivatable mCherry (PAmCherry) protein. The shells of such encapsulins were modified using chemical conjugation of human transferrin (Tf) prelabeled with a fluorescein-6 (FAM) maleimide acting as a vector. We demonstrate that the vectorized encapsulin specifically binds to transferrin receptors (TfRs) on the membranes of mesenchymal stromal/stem cells (MSCs) followed by internalization into cells. Two spectrally separated fluorescent signals from Tf-FAM and PAmCherry are clearly distinguishable and co-localized. It is shown that Tf-tagged Mx encapsulins are internalized by MSCs much more efficiently than by fibroblasts. It has been also found that unlabeled Tf effectively competes with the conjugated Mx-Tf-FAM formulations. That indicates the conjugate internalization into cells by Tf-TfR endocytosis pathway. The developed nanoplatform can be used as an alternative to conventional nanocarriers for targeted delivery of, e.g., genetic material to MSCs.

## Impact statement

The paper is dedicated to engineering of a novel genetically encoded vectorized encapsulin-based nanocontainer system. Encapsulin shells are extremely robust structures, resistant to high temperature and denaturation, and they also protect the payloads inside the shell reliably. Encapsulins also have very developed surface area which allows both loading various molecules inside and functionalizing the shells using vector groups. Therefore, encapsulins are very promising to serve as selective targeting nanocarriers. The data obtained allowed us to conclude that the interaction between Mx-Tf-FAM and MSCs was associated with Tf-TfR internalization pathway.

## Introduction

Mesenchymal stromal/stem cells (MSCs) are cells capable of mesodermal cell lineages differentiation. These cells can differentiate into osteocytes, chondrocytes, adipocytes, and muscle cells [1]. The possibility of obtaining MSCs from different biological niches, their low immunogenic and modulating features [2] enable autologous and allogeneic transplantation of these cells. MSCs are employed for the treatment of various pathologies such as bone and cartilage diseases [3, 4], cardiovascular diseases [5, 6], neurological disorders [7–9], bronchial diseases [10], and many others. Despite some success of therapy with MSCs, the clinical application of these cells is restricted by the phenotypic plasticity. These cells lose stemness *ex vivo*, resulting in reduced therapeutic potential [11]. Various genetic modifications of MSCs can partially overcome this issue.

It is considered that MSCs transduction using, for example, lentiviral vectors is quite effective. However, this method of cell modification is not always applicable due to safety concerns associated with the possibility of insertional mutagenesis, as well as possible immunogenicity of viral antigens [12]. In addition, lentiviral gene delivery systems have a relatively small transgene cargo capacity [13].

There are also numerous methods of nonviral gene delivery to MSCs. Some approaches are realized by violating the integrity of cell membranes as a result of microinjection or electroporation [14, 15], or using cell penetrating peptide [16]. Other methods are based on the use of various nanocarriers, such as inorganic materials, lipids, polymers, etc., [17, 18]. These techniques are safer, but, with rare exceptions, may cause a decrease in cell viability [19]. Therefore, the development of new gene delivery tools, devoid of the described disadvantages, is an extremely urgent task. Here, we describe a new targeted delivery system that employ the *Myxococcus xanthus* (Mx) encapsulins as vector nanocontainers. Encapsulins are bacterial capsid-like, high molecular weight structures consisting of a protein shell and a cargo protein contained within. The encapsulin shells are extremely stable, have different diameters, and reliably protect the cargo proteins. The proteins carried by encapsulins are diverse and differ in their functions [20]. Since the discovery of encapsulins in 1994, the focus of their investigation has gradually shifted towards using these structures as nanocontainer systems. A number of encapsulin-based delivery systems were developed. For example, *Thermotoga maritima* (*T. maritima*) encapsulin-based system that selectively binds glucose-regulated protein 78 in human HepG2 carcinoma cells [21]. A similar system was developed for targeted delivery of encapsulins loaded with mini SOG (mini-Singlet Oxygen Generator) to HER2^+^ breast cancer cells [22]. A possibility of RNA or DNA loading into encapsulin shells was also demonstrated in recent works [23, 24].

The wild type Mx encapsulin (Supplementary Figure S1) consists of a protein shell (EncA) self-assembled of 180 identical monomeric proteins (32.5 kDa each) confining three ferritin-like cargo proteins (EncB, 17 kDa; EncC, 13 kDa; EncD, 11 kDa) [25]. We replaced Mx EncBCD ferritin-like native cargo with an irreversibly photoactivatable derivative of mCherry (PAmCherry) fluorescent protein as we previously described [26]. We further modified the Mx shells with human transferrin (Tf) preliminary labeled with a fluorescent dye of fluorescein-6 (FAM) maleimide to provide selective binding to the transferrin receptors (TfRs) on MSCs. The binding selectivity of the resulting vector system (Mx-Tf-FAM) to MSCs was confirmed by the laser scanning confocal microscopy and imaging flow cytometry. It was also demonstrated that free Tf competed for binding with the Mx-Tf-FAM conjugates. That indicated the conjugate internalization into the cells by receptor-mediated endocytosis.

The current study demonstrates a biodegradable, non-toxic and non-viral nanocarrier system that selectively binds to TfRs on the membranes of MSCs followed by rapid internalization into cells via Tf-TfR pathway. Such Tf-mediated targeting may be useful not only for the delivery of genetic material to MSCs, but also for therapeutic purposes in the treatment of cancer. For example, to deliver siRNA (small interfering RNA) to TfRs overexpressing tumor cells.

## Materials and methods

### Cell cultures

The procedures performed with patient tissue samples were in accordance with the Declaration of Helsinki and approved by the Local Ethics Committee, Medical Research, and Education Center, Lomonosov Moscow State University (IRB00010587), protocol #4 (2018). Samples of human adipose-derived MSCs were collected from the Cryobank of the Institute for Regenerative Medicine of Lomonosov Moscow State University (collection ID MSC_AD_MSU^1^, accessed on 17 November 2023). Informed consent was obtained from all subjects involved in the study.

MSCs were cultured on DMEM/F12 supplemented with 10% FBS (HyClone, Cytiva, Washington, D.C., United States), 2 mM L-glutamine, and antibiotics (100 U/mL penicillin, 0.1 mg/mL streptomycin) in T-75 cell culture flasks. Human fibroblasts were kindly provided by the Laboratory of Cell Biology of Lopukhin Center of Physical-Chemical Medicine of FMBA of Russia. 293T EncA_PAmCherry cells and fibroblasts were cultured in DMEM in T-25 flasks with addition of 100 U/mL penicillin, 0.1 mg/mL streptomycin, 2 mM L-glutamine and 10% FBS. All cell cultures were grown under standard conditions (5% CO_2_ and 37°C). All reactants were purchased from Gibco (New York, NY, United States) and laboratory plastic was purchased from Corning (New York, NY, United States).

### Mesenchymal stem cells differentiation

MSCs differentiation into chondroblasts, adipocytes, and osteoblasts was carried out using a commercial StemPro^®^ Differentiation kit according to the manufacturer’s instruction [27–29]. After the differentiation was completed, the resulting chondroblasts, adipocytes, and osteoblasts, and were stained using Alcian Blue (Sigma-Aldrich, St. Louis, MI, United States), OilRed O (Sigma-Aldrich), and Alizarin Red (Sigma-Aldrich), respectively. A Primo Vert light inverted microscope (Zeiss, Baden-Württemberg, Germany) was used to obtain images of the stained cells.

### Flow cytometry

For the flow cytometry, MSCs were labeled with conjugated primary antibodies for MSCs positive (CD105, CD73, and CD90) and negative (CD19, CD45, CD34, CD14, and HLA-DR) markers using the MSC Phenotyping Cocktail Kit, anti-human (Miltenyi Biotec, Bergisch Gladbach, and Germany) according to the standard protocol [30]. The expression of markers was assessed with a flow cytometer-sorter (FACS Aria III, BD Biosciences, Franklin Lakes, NJ, USA). Dead cells were excluded from the analysis by staining with SytoxBlue Dead Cell stain (Invitrogen, Waltham, MA, United States); cell debris, and duplexes were excluded based on the forward and side light scattering parameters.

### Imaging flow cytometry

24 h prior to the experiment MSCs were cultured in a 96-well plate (∼7.000 cells/well, Corning) in 100 µL of DMEM/F12 medium supplemented with 10% FBS. For Tf competition binding assay, Mx-Tf-FAM encapsulins in PBS (50 ng/μL) mixed with free soluble Tf (in fivefold excess) were added to the cells. After 1.5 h incubation the cells were washed three times with DPBS to remove the unbound Mx-Tf-FAM and Tf. Next, the cells were detached from the plastic, precipitated (500× g, 5 min), and resuspended in 0.1 mL of 4% PFA in PBS (pH 7.4). Cytometric assays were performed using an ImageStream X Mark II imaging flow cytometer (Amnis, Luminex Corporation, Austin, TX, United States), which is a powerful tool for investigation of binding [31, 32]. The present studies were carried out using a ×40 objective, 488‐nm (175 mW) laser for fluorescence excitation, and at 785‐nm laser light (0.5 mW) for side scatter measurements.

### Laser scanning confocal microscopy

The cell imaging was performed using an A1R MP+ (Nikon, Tokyo, Japan) instrument (405 and 561 nm laser wavelengths, oil immersion objective lenses Apo TIRF 60×/1.49) or Eclipse Ti2 (Nikon, Tokyo, Japan) (405, 561, and 642 nm laser wavelengths, Apo 25×/1,1 water immersion objective lenses) laser scanning confocal microscopes. The scanning was performed using the ThorImageLS 2.4 Software (Thorlabs, Newton, NJ, United States) and Nikon NIS elements 4.50 software (Nikon, Tokyo, Japan). To process the images the ImageJ2 Fiji software was used.

### Immunoprecipitation

293T EncA_PamCherry cells were placed into 6-wells plates in 2 mL of DMEM culture medium (1.5 × 10^6^ cells/well), FLAG-tagged Mx encapsulins were isolated from 293T EncA_PamCherry cells after 24 h of cultivation using Anti-FLAG M2 Affinity gel (Sigma Aldrich) according to the manufacturer’s instruction as described earlier [26].

### Dynamic light scattering

The hydrodynamic diameter of purified Mx encapsulins was measured via ZetaSizer Nano ZS (Malvern, UK) at 25°C using standard glass cuvettes containing 1,000 μL of eluate solution in TBS according to a procedure recommended the manufacturer [33].

### Western blot analysis

Briefly, 293T EncA_PamCherry cells were, and the resulting lysate was centrifuged for 20 min at 14,000 × g. Buffer 5× was added to various amounts (2 μL, 5 μL, 10 μL, and 20 µL) of the cell lysate, then heated at 95°C, and after that cooled on ice. The lysate was loaded onto gel and electrophoresed for 25 min at 80 V and then for 1.5 h at 100 V. The gel was moved into a transfer buffer. The activated nitrocellulose membrane was placed over the gel. The transfer procedure was fulfilled for 1 h at 100 V in a chamber filled with transfer buffer. The membrane was thoroughly washed in PBST to remove the transfer buffer. The membrane was incubated with 5% non-fat milk in PBST solution for 2 h to prevent nonspecific binding and then washed again. The membrane was incubated with anti-Flag Tag antibodies (1:1,000, BioLegend, San Diego, United States) for 2 h followed by washing three times with DPBS. Next, secondary antibodies (1:1,000, goat anti-mouse IgG, Santa Cruz Biotechnology, Dallas, United States) conjugated with alkaline horseradish peroxidase were added. The results were readout by a ChemidocMP Imaging system (BioRad, Hercules, United States).

### Conjugation with fluorescent dye and engineering of Tf-FAM labeled Mx encapsulins

At first, apo transferrin (10 mg, Merck, Rahway, NJ, United States) was dissolved in 2 mL of DPBS (pH = 6.8). Fluorescein-6 maleimide (Lumiprobe, Russia) in the amount of 2 µL (20 mg/mL in DMSO) was added and mixed overnight at constant stirring at ambient temperature. After that, the resulting solution was purified five times using centrifugal concentrator 30 kDa cut off filters (Millipore Billerica, United States) and adjusted to 1 mL in PBS (pH = 7.4). Then, 10 μL of 6-Maleimidohexanoic acid N-hydroxysuccinimide ester at concentration of 0.5 mg/mL in water:DMSO 1:1 was mixed with Tf-FAM followed by 1 h incubation at ambient temperature. The solution was also purified five times by 30 kDa cut off filters and resolved in 1 mL PBS (pH = 6.8). At last, the isolated Mx encapsulins were also diluted in PBS at pH = 6.8 and mixed 1:1: by weight in 1 mL of the reaction mixture overnight. The produced conjugates were washed seven times using 100 kDa cut off filters and PBS (pH = 7.4). The 500 ng/μL Mx-Tf-FAM stock solution was kept at 4°C.

### Cellular uptake of Mx and Mx-Tf-FAM encapsulins

MSCs and human fibroblasts were seeded on a 2-well µ-Dish for confocal microscopy (Ibidi, Martinsried, Germany) in the amount of 1 × 10^4^ cells/dish and cultured for 24 h prior the assay. Following the cultivation, 30 μL of Mx or Mx-Tf-FAM samples in PBS aligned according to protein concentration was added to the cells to the final concentration of ∼290 ng/μL. The cells were incubated at 5% CO_2_ and 37°C for 30 and 90 min. After that, the culture medium was removed. The cells were washed thoroughly with DPBS to remove unbounded encapsulins. The cells were studied using a Nikon Eclipse Ti2 confocal microscope.

## Results

### Characterization of MSCs

**FIGURE 1:**
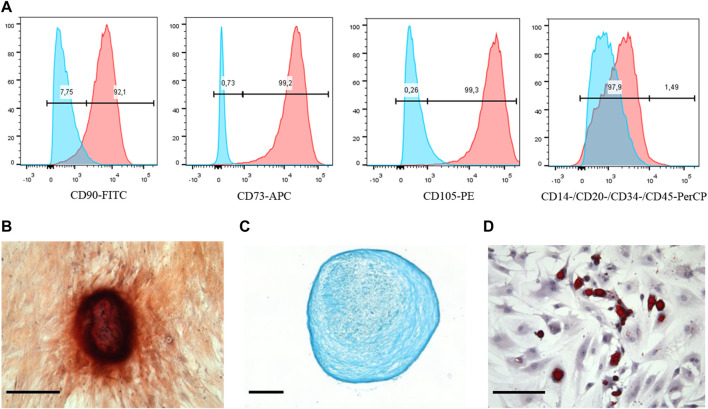
Phenotypic characterization and multilineage differentiation of human MSCs. **(A)** Flow cytometry analysis of the MSCs surface markers expression: red graphs show experimental samples, while blue graphs—negative control. MSCs differentiation: **(B)** Osteogenic revealed by Alizarin Red, **(C)** chondrogenic revealed by Alcian Blue, and **(D)** adipogenic revealed by Oil Red staining. Scale bars are 50 μm.

At first, the markers on the cell surface were detected using the flow cytometry. The data obtained showed that the expression profile of CD markers was typical for MSCs. The cells were positive for the main MSCs markers (CD73, CD90, and CD105) and negative for such markers as CD14, CD20, CD34, and CD45 (Figure 1A). Besides, it is well-known that MSCs have a potential for multidirectional differentiation into a different cell type (chondrocytes, adipocytes and osteoblasts). We used commercially available kits that allowed implementation of *in vitro* MSCs differentiation. As can be seen in Figure 1B, MSCs exhibited positive Alizarin Red staining after osteogenic induction; positive Alcian blue (as shown in Figure 1C) and Oil Red O (as demonstrated in Figure 1D) staining after chondrogenic and adipogenic induction, respectively. Thus, the mesenchymal identity (i.e., the ability for multilineage differentiation and CD markers expression profile) of MSCs was confirmed. The images of the negative control cells for each staining are demonstrated in Supplementary Figure S2.

### Isolation and characterization of Mx encapsulins

Our previous study showed that expression of stable Mx encapsulin protomer protein such as EncA tagged with a DYKDDDDK sequence and the fluorescent PAmCherry cargo encoding genes could be successfully achieved in mammalian 293T cells. We also demonstrated that encapsulated label did not affect cell proliferation and viability [26].

**FIGURE 2:**
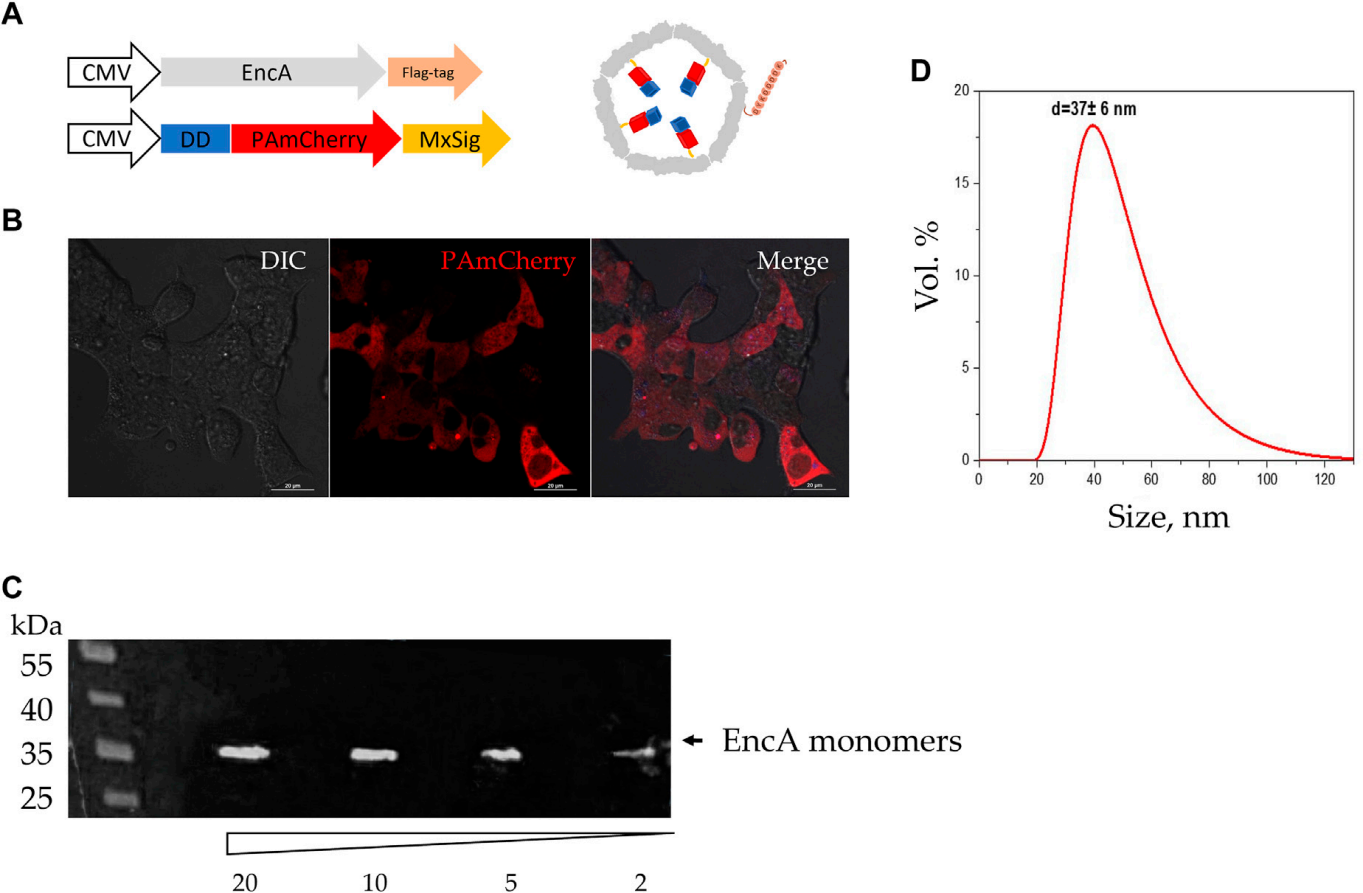
Assembly of Mx encapsulins and PAmCherry photoactivation in 293T cells. **(A)** Schematic representation of the genetic constructs (with minor omissions) and the assembled Mx encapsulin shell containing a photoactivatable protein. At the N-terminus, PAmCherry is fused to a destabilization domain (DD) to degrade such a protein that is not encapsulated within the envelope. MxSig is C-terminal Mx encapsulation signal, CMV—cytomegalovirus promoter; **(B)** 293T EncA_PAmCherry cells after irradiation with a 405 nm laser followed by excitation with light with 561 nm wavelength. Laser scanning confocal microscopy using Nikon A1 MP instrument, scale bars are 20 μm; DIC—differential interference contrast; **(C)** Western blot analysis against FLAG-tag on EncA protomer proteins; the numbers indicate the volume of cell lysate (2, 5, 10, and 20 µL) added to the gel; the triangle indicates a decrease in the volume of cell lysate added to the gel; black arrow indicates a band with a molecular weight of 35 kDa region; **(D)** Dynamic light scattering analysis of isolated Mx encapsulins (d = 37 ± 6 nm; PDI 0.4).

In this study we first verified the presence of necessary transgenic sequences, as well as proteins translated from them. For this purpose, PAmCherry was photoactivated in the transgenic 293T cells. PAmCherry encoding gene is coupled to a short protein (Figure 2A) unstable under physiological conditions [destabilization domain (DD)] [34]. The presence of DD leads to PAmCherry protein degradation by the proteasome if not encapsulated into the Mx shell. As can be seen from Figure 2B, after the activation with a laser with the wavelength of 405 nm and subsequent excitation with a 561 nm light, a bright red fluorescent signal from PAmCherry was detected in the cell cytoplasm. Therefore, we could conclude that the expression of encapsulated PAmCherry label was retained. The protein expression of EncA protomers was also confirmed by the Western blot analysis. A single band of protomer protein with a weight of ∼35 kDa was clearly visible (Figure 2C). The encapsulins were further isolated from the cells, and the hydrodynamic size of the shell was measured using the DLS analysis. According to the DLS analysis, the hydrodynamic size of isolated Mx encapsulins (Figure 2D) was 37 ± 6 nm with 0.4 PDI (polydispersity index). This value corresponds well to the size of the Mx encapsulin shell described in literature [35].

### Engineering of Tf-FAM double-labeled Mx encapsulins

**FIGURE 3:**
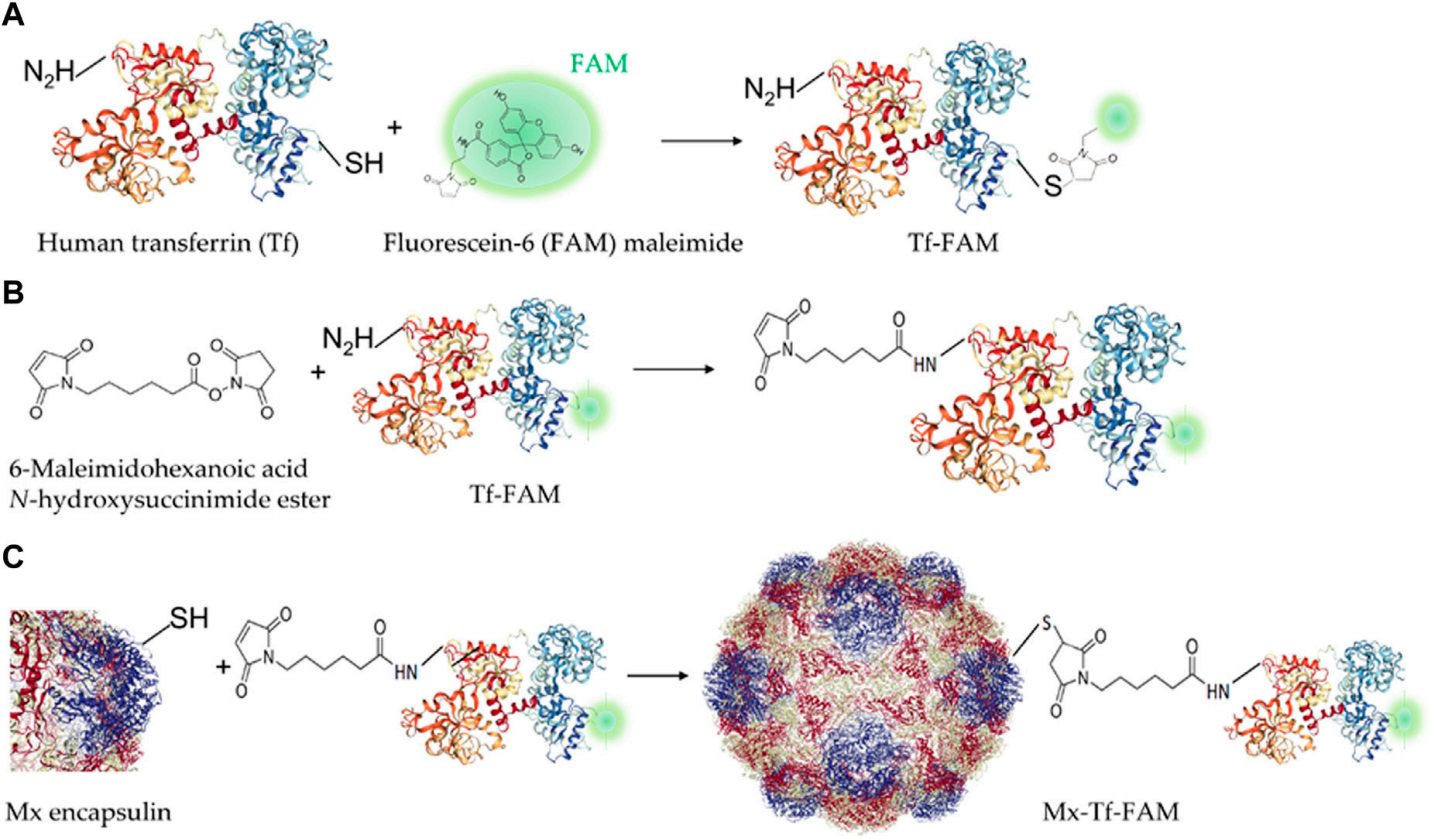
Schemes of Tf-FAM-conjugated Mx encapsulins preparation. **(A)** Labeling of human Tf with FAM; **(B)** Tf-FAM binding with the bifunctional EMCS linker; **(C)** Conjugation of Tf-FAM with Mx encapsulin using EMCS.

We have developed a strategy for FAM-labeled Tf conjugation with Mx encapsulin shells using EMCS − a heterobifunctional cross-linking reagent with amine and sulfhydryl reactivity (Figure 3). Thus, the receptor-targeted Mx-Tf-FAM obtained in this study has two fluorescent labels: PAmCherry (spectral maximum of exaction at 564 nm and emission at 595 nm) inside the shell, and FAM (spectral maximum of exaction at 492 nm and emission at 517 nm) on the shell. These two fluorescent labels, on the one hand, enable verification of Tf-FAM localization on the shells of Mx encapsulins, and, on the other hand, visualize the conjugate internalization into cells.

### Cellular uptake of vectorized Mx encapsulins by MSCs

**FIGURE 4:**
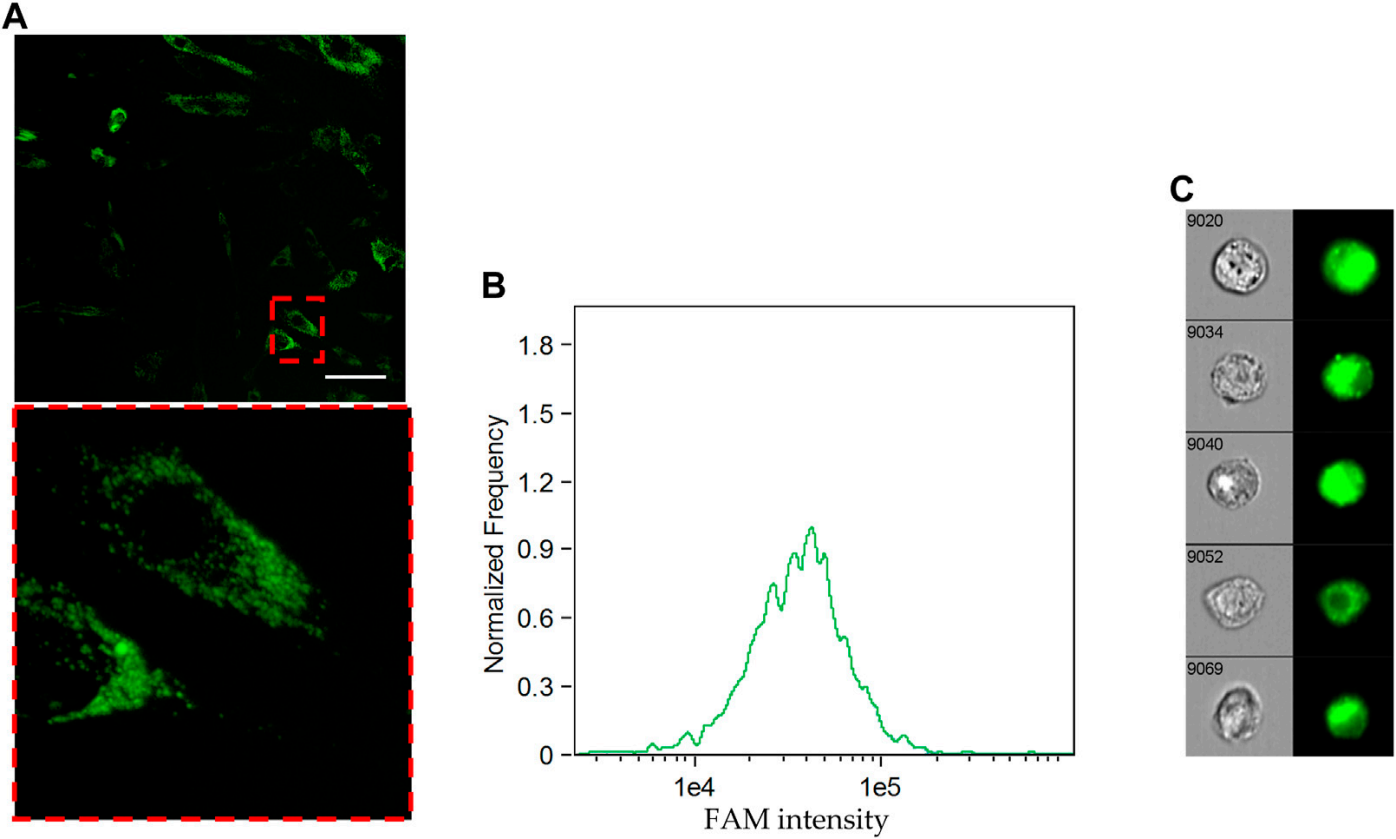
Cellular uptake of FAM-labeled transferrin by MSCs. **(A)** Uptake of Tf-FAM in MSCs after 90 min incubation. Green fluorescent signal shows FAM. Laser scanning confocal microscopy, scale bar 50 μm. **(B)** FAM intensity histogram and **(C)** representative images of Tf-FAM labeled MSCs in bright field and FITC channels of an imaging flow cytometer.

First of all, the ability of Tf-FAM to bind to TfRs was analyzed by the laser scanning confocal microscopy using MSCs and human fibroblasts. The obtained microphotographs (Figure 4A) show that TfRs are visualized as characteristic placoids with cytoplasmic localization. The imaging flow cytometry measurements (Figures 4B, C) also confirmed MSCs to be TfR positive. It can be seen that the TfR expression in fibroblasts is dramatically lower compared to that of MSCs (Supplementary Figure S3).

**FIGURE 5:**
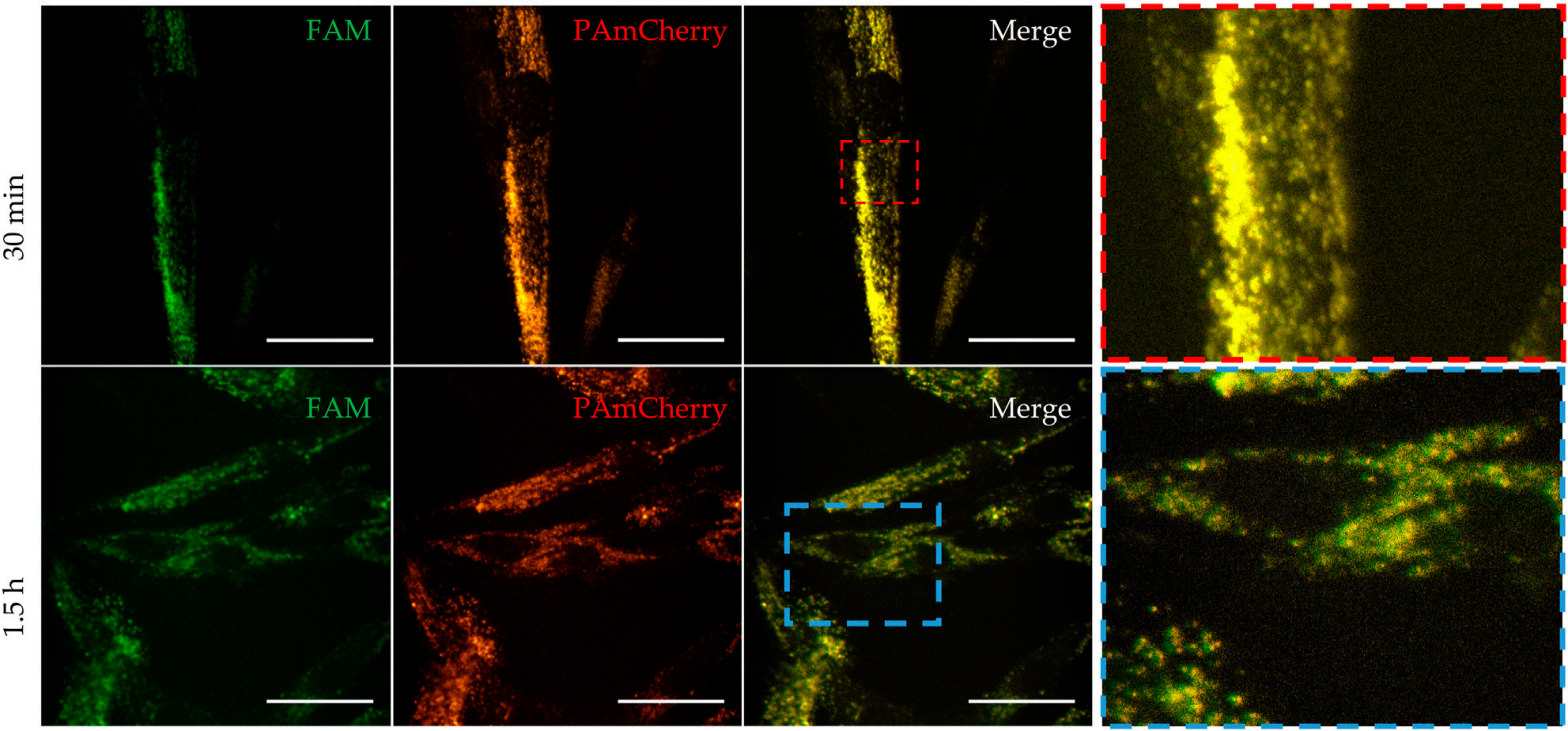
Uptake and intracellular localization of Mx-Tf-FAM in MSCs after 30 min (upper row) and after 90 min (lower row) of incubation measured by confocal microscopy. Green fluorescent signal—FAM, red fluorescent signal—PAmCherry, scale bars are 50 μm.

Next, the laser scanning confocal microscopy was used for qualitative estimation of the uptake and intracellular distribution of Mx-Tf-FAM by MSCs. The obtained micrographs (Figure 5) clearly show the co-localization of two intense fluorescent signals in green and red spectral ranges. It can also be seen that the fluorescent signal is localized predominantly on membranes of the cells after 30 min of incubation (Figure 5, upper row), and after 1.5 h of incubation (Figure 5, lower row), the signal from the labels is also observed in the cytoplasm of the cells. The uptake of Mx-Tf-FAM by control fibroblasts is presented in Supplementary Figure S4. In contrast to the result obtained for MSCs, the fluorescent signals from Tf-FAM and PAmCherry in fibroblasts are barely distinguishable.

**FIGURE 6:**
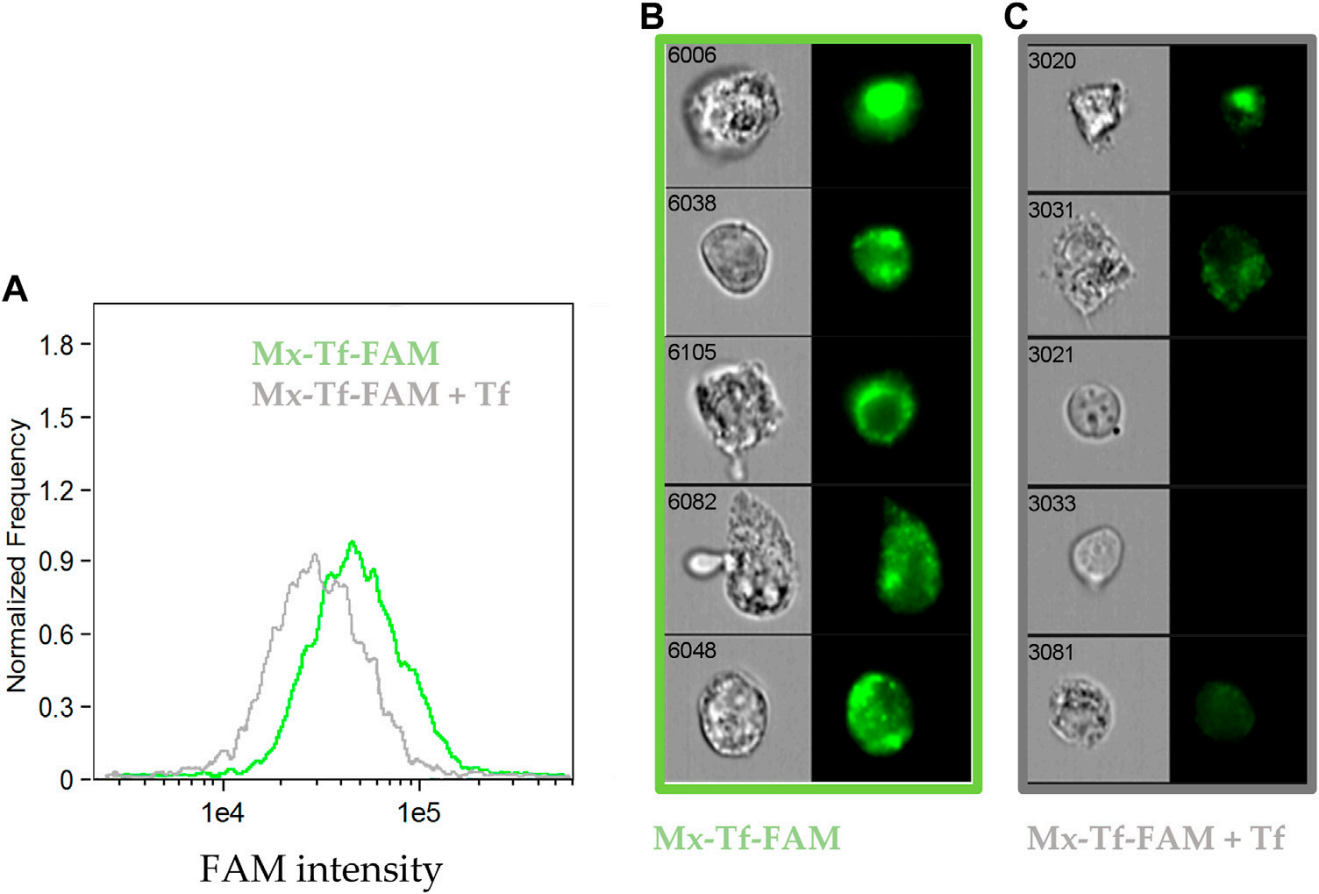
The ability of free unlabeled Tf to compete for binding with Mx-Tf-FAM assessed by the imaging flow cytometry. **(A)** FAM intensity histograms for MSCs labeled with Mx-Tf-FAM and under incubation in an excess of free unlabeled Tf that inhibit further binding of Mx-Tf-FAM; **(B)** representative images of Mx-Tf-FAM labeled MSCs in bright field and FITC channels **(C)** representative images of MSCs labeled with Mx-Tf-FAM in an excess of free Tf in bright field and FITC channels.

At last, to verify that the interaction between Mx-Tf-FAM and MSCs was related to Tf-TfR interaction, we assessed by the imaging flow cytometry the ability of free Tf to compete for interaction with the vectorized encapsulins. It was shown (Figure 6A) that the median intensity in the “Mx-Tf-FAM” sample exceeded that of the “Mx-Tf-FAM + Tf” sample. It can also be seen (Figure 6B) that the FAM fluorescence intensity in the “Mx-Tf-FAM” sample is significantly higher compared to that observed in the “Mx-Tf-FAM + Tf” sample (Figure 6C). In addition, it can be noted that in spite of MCSs presence in bright field, the FAM signal is almost absent (Figure 6C).

The results obtained demonstrated that the unlabeled Tf effectively competed with the conjugated Mx-Tf-FAM formulations. That indicates conjugate internalization into cells by, supposedly, receptor-mediated endocytosis. Certainly, further research is required to recognize the exact Mx-Tf-FAM endocytosis pathway, for example, using endocytosis blockers.

## Discussion

Over the past decades, many different nanocarrier delivery systems were developed, e.g., gold [16, 36] and iron oxide [37, 38] nanoparticles, including those with metal-organic framework storages for drug and *in vivo* gene delivery [39, 40]; micelles [41]; hybrid liposome-, polymer- [42], and protein-based nanoparticles [43, 44]. The shells of such carriers can be modified by adding to their surface various receptor molecules to provide fast binding kinetics to the related ligand [45] and achieve specific targeting [46]. An important part of these studies was investigation of toxicity of the obtained nanocarriers and the products of their *in vivo* biodegradation [47–49]. Among others, the protein-based nanocontainers (virus-like particles, ferritins, encapsulins) are of particular interest due to their stability, non-toxicity, and biodegradability.

As it is mentioned above, encapsulins are very similar in structure to viral capsids. Like viral capsids, they reliably keep the internal payload of the shell. In our recent work [26] on RAW 264.7 cells, it was shown that Mx encapsulin shells protect the cargo protein from the action of intracellular proteases for at least 2 h. Interestingly, there is one more research, in which the authors investigated the uptake of fluorescently labeled encapsulins by macrophages (J774 macrophages cell line) [50] but they used a significantly longer internalization time.

In the present research, FAM-labeled human transferrin was obtained by binding of SH-groups with FAM-maleimide. Tf-FAM was then chemically conjugated using a linker bound with NH2-groups of Tf-FAM and SH-groups on the encapsulin shells. According to the PDB data (entry 7S20), each EncA monomer has two cysteines in its structure. In total, the Mx encapsulin shell consists of 180 such monomers so the EMCS linker is potentially capable of binding FAM-labeled transferrin in 360 sites. That enables amplification of the fluorescent signal of FAM.

Prior to exploring the Tf-mediated targeting, we tested the uptake of non-vector Mx encapsulins by MSCs and human fibroblasts. No, even minimal, fluorescent signal of PAmCherry was detected in the cells after 2 h of incubation (Supplementary Figure S5). Unlike macrophages, neither MSCs nor fibroblasts are professional phagocytes. That is why they are not able to rapidly internalize protein structures of high molecular weight. The fact that Mx encapsulins are naturally inert to MSCs again confirms the need for further development of the targeted nanocarriers.

TfR-mediated endocytosis is a relatively fast process but the rate of Tf internalization varies in different cells [51]. In the case of MSCs, internalization of Mx-Tf-FAM conjugates could be registered after 30 min of incubation, while after 1.5 h incubation, fluorescent signals from FAM and PAmCherry were detected throughout the cell cytoplasm.

The targeted delivery system presented in this work is effective not only for MSCs but also for other cells of high TfR expression level. This is particularly true for malignant cells. It is well known that TfR plays an important role in the processes of proliferation, migration, and invasion of cancer cells [52–54]. As an example, one such neoplasm is known to be human glioblastoma multiforme—the primary grade IV brain tumor in adults with poor prognosis [55]. The model PAmCherry cargo protein used in this study can be further replaced/supplemented with different genetic material [56, 57], and the resulting nanoplatform can be an alternative to viral vectors for nucleic acids delivery to MSCs [58]. Moreover, this platform is suitable for intracellular delivery of recombinant proteins which is a potential strategy against a wide range of diseases.

Regarding the use of encapsulin-based nanocontainer systems for *in vivo* studies, it is important to address the issue of potential immunogenicity of the xenogeneic proteins. We have already discussed this problem partially: we presently know that, at least, *Quasibacillus thermotolerans* encapsulin-containing cells do not cause an immune response when implanted into mice [59] and rats [60]. In addition, an article was recently published evaluating *in vivo* behavior of *Thermotoga maritima* encapsulins administered intravenously into mice [61]. The work demonstrated that IV-injected *Thermotoga maritima* encapsulins exhibited an excellent safety profile. The results obtained suggest that encapsulins from bacteria of other strains may also be safe for *in vivo* use.

It is noteworthy that all the described encapsulin-based delivery systems used encapsulins purified from bacteria of various strains. We have shown a possible alternative approach to encapsulin isolation using an eukaryotic cell line. We hope that our results will facilitate further developments of similar techniques.

## Conclusion

Undoubtedly, encapsulins currently remain a relatively new object of research; their properties and their original functions in prokaryotes should be comprehensively studied. However, practical applications of encapsulins in the field of biotechnology is becoming increasingly promising. In this study, we describe a new vector tool for delivery of nucleic acids to mesenchymal stem cells. The entire process for obtaining of Mx-Tf-FAM conjugates was carried out under completely aseptic conditions, and that enabled development of a sterile and endotoxin-free delivery system targeted by transferrin receptors. The vectorized Mx encapsulins containing a PAmCherry label can bind transferrin receptors on the surface of mesenchymal stem cells followed by internalization into cells by, presumably, receptor-mediated endocytosis, while maintaining the PAmCherry and FAM fluorescent signals.

Summarizing, compared to nanoplatforms based on inorganic and/or polymer nanoparticles encapsulin-based nanocontainer systems have a number of advantages. These structures are not only extremely robust. These are completely biodegradable, non-toxic, endotoxin-free protein nanoparticles. The synthesis of encapsulins is genetically based, which, on the one side, means its reproducibility is extremely high and, on the other side, there is a possibility for genetic manipulation. Thus, these features of encapsulins promise significant customization flexibility and broad usability. The obtained nanocarrier can be supplemented not only with nucleic acids, but also with peptides or therapeutic agents for specific delivery to MSCs.

## Data Availability

The original contributions presented in the study are included in the article/Supplementary Material, further inquiries can be directed to the corresponding author.

## Footnotes

1
www.human.depo.msu.ru

## References

[1] Weatherall EL Avilkina V Cortes-Araya Y Dan-Jumbo S Stenhouse C Donadeu FX Differentiation potential of mesenchymal stem/stromal cells is altered by intrauterine growth restriction. Front Vet Sci (2020) 7:558905. 10.3389/fvets.2020.558905 33251256 PMC7676910

[2] Heo JS Choi Y Kim HS Kim HO. Comparison of molecular profiles of human mesenchymal stem cells derived from bone marrow, umbilical cord blood, placenta and adipose tissue. Int J Mol Med (2016) 37:115–25. 10.3892/ijmm.2015.2413 26719857 PMC4687432

[3] Lee WS Kim HJ Kim KI Kim GB Jin W. Intra-articular injection of autologous adipose tissue-derived mesenchymal stem cells for the treatment of knee osteoarthritis: a phase IIb, randomized, placebo-controlled clinical trial. Stem Cells Translational Med (2019) 8:504–11. 10.1002/sctm.18-0122 PMC6525553 30835956

[4] Zhao X Ruan J Tang H Li J Shi Y Li M Multi-compositional MRI evaluation of repair cartilage in knee osteoarthritis with treatment of allogeneic human adipose-derived mesenchymal progenitor cells. Stem Cell Res Ther (2019) 10:308. 10.1186/s13287-019-1406-7 31639063 PMC6805685

[5] Bolli R Perin EC Willerson JT Yang PC Traverse JH Henry TD Allogeneic mesenchymal cell therapy in anthracycline-induced cardiomyopathy heart failure patients: the CCTRN SENECA trial. JACC: CardioOncology (2020) 2:581–95. 10.1016/j.jaccao.2020.09.001 33403362 PMC7781291

[6] He X Wang Q Zhao Y Zhang H Wang B Pan J Effect of intramyocardial grafting collagen scaffold with mesenchymal stromal cells in patients with chronic ischemic heart disease: a randomized clinical trial. JAMA Netw Open (2020) 3:e2016236. 10.1001/jamanetworkopen.2020.16236 32910197 PMC7489863

[7] Uccelli A Laroni A Brundin L Clanet M Fernandez O Nabavi SM MEsenchymal StEm cells for Multiple Sclerosis (MESEMS): a randomized, double blind, cross-over phase I/II clinical trial with autologous mesenchymal stem cells for the therapy of multiple sclerosis. Trials (2019) 20:263. 10.1186/s13063-019-3346-z 31072380 PMC6507027

[8] Petrou P Kassis I Levin N Paul F Backner Y Benoliel T Beneficial effects of autologous mesenchymal stem cell transplantation in active progressive multiple sclerosis. Brain (2020) 143:3574–88. 10.1093/brain/awaa333 33253391

[9] Namestnikova DD Gubskiy IL Revkova VA Sukhinich KK Melnikov PA Gabashvili AN Intra-arterial stem cell transplantation in experimental stroke in rats: real-time MR visualization of transplanted cells starting with their first pass through the brain with regard to the therapeutic action. Front Neurosci (2021) 15:641970. 10.3389/fnins.2021.641970 33737862 PMC7960930

[10] Powell SB Silvestri JM. Safety of intratracheal administration of human umbilical cord blood derived mesenchymal stromal cells in extremely low birth weight preterm infants. J Pediatr (2019) 210:209–13.e2. 10.1016/j.jpeds.2019.02.029 30992220

[11] Yang YHK Ogando CR Wang See C Chang TY Barabino GA. Changes in phenotype and differentiation potential of human mesenchymal stem cells aging *in vitro* . Stem Cell Res Ther (2018) 9:131. 10.1186/s13287-018-0876-3 29751774 PMC5948736

[12] Oggu GS Sasikumar S Reddy N Ella KKR Rao CM Bokara KK. Gene delivery approaches for mesenchymal stem cell therapy: strategies to increase efficiency and specificity. Stem Cell Rev Rep (2017) 13:725–40. 10.1007/s12015-017-9760-2 28815481

[13] Kalidasan V Ng WH Ishola OA Ravichantar N Tan JJ Das KT. A guide in lentiviral vector production for hard-to-transfect cells, using cardiac-derived c-kit expressing cells as a model system. Sci Rep (2021) 11:19265. 10.1038/s41598-021-98657-7 34584147 PMC8478948

[14] Tsulaia TV Prokopishyn NL Yao A Carsrud NV Carou MC Brown DB Glass needle-mediated microinjection of macromolecules and transgenes into primary human mesenchymal stem cells. J Biomed Sci (2003) 10:328–36. 10.1159/000070098 12711860

[15] Cervia LD Chang CC Wang L Mao M Yuan F. Enhancing electrotransfection efficiency through improvement in nuclear entry of plasmid DNA. Mol Ther - Nucleic Acids (2018) 11:263–71. 10.1016/j.omtn.2018.02.009 29858061 PMC5992438

[16] Elizarova TN Antopolsky ML Novichikhin DO Skirda AM Orlov AV Bragina VA A straightforward method for the development of positively charged gold nanoparticle-based vectors for effective siRNA delivery. Molecules (2023) 28:3318. 10.3390/molecules28083318 37110552 PMC10144622

[17] Gonzalez-Fernandez T Sathy BN Hobbs C Cunniffe GM McCarthy HO Dunne NJ Mesenchymal stem cell fate following non-viral gene transfection strongly depends on the choice of delivery vector. Acta Biomater (2017) 55:226–38. 10.1016/j.actbio.2017.03.044 28363788

[18] Sizikov AA Nikitin PI Nikitin MP. Magnetofection *in vivo* by nanomagnetic carriers systemically administered into the bloodstream. Pharmaceutics (2021) 13:1927. 10.3390/pharmaceutics13111927 34834342 PMC8619128

[19] Stewart MP Langer R Jensen KF. Intracellular delivery by membrane disruption: mechanisms, strategies, and concepts. Chem Rev (2018) 118:7409–531. 10.1021/acs.chemrev.7b00678 30052023 PMC6763210

[20] Gabashvili AN Chmelyuk NS Oda VV Leonova MK Sarkisova VA Lazareva PA Magnetic and fluorescent dual-labeled genetically encoded targeted nanoparticles for malignant glioma cell tracking and drug delivery. Pharmaceutics (2023) 15:2422. 10.3390/pharmaceutics15102422 37896182 PMC10609955

[21] Moon H Lee J Min J Kang S. Developing genetically engineered encapsulin protein cage nanoparticles as a targeted delivery nanoplatform. Biomacromolecules (2014) 15:3794–801. 10.1021/bm501066m 25180761

[22] Van de Steen A Khalife R Colant N Mustafa Khan H Deveikis M Charalambous S Bioengineering bacterial encapsulin nanocompartments as targeted drug delivery system. Synth Syst Biotechnol (2021) 6:231–41. 10.1016/j.synbio.2021.09.001 34541345 PMC8435816

[23] Kwon S Giessen TW. Engineered protein nanocages for concurrent RNA and protein packaging *in vivo* . ACS Synth Biol (2022) 11:3504–15. 10.1021/acssynbio.2c00391 36170610 PMC9944510

[24] Almeida AV Carvalho AJ Calmeiro T Jones NC Hoffmann SV Fortunato E Condensation and protection of DNA by the Myxococcus xanthus encapsulin: a novel function. Int J Mol Sci (2022) 23:7829. 10.3390/ijms23147829 35887179 PMC9321382

[25] McHugh CA Fontana J Nemecek D Cheng N Aksyuk AA Heymann JB A virus capsid-like nanocompartment that stores iron and protects bacteria from oxidative stress. EMBO J (2014) 33:1896–911. 10.15252/embj.201488566 25024436 PMC4195785

[26] Gabashvili AN Chmelyuk NS Sarkisova VA Melnikov PA Semkina AS Nikitin AA Myxococcus xanthus encapsulin as a promising platform for intracellular protein delivery. Int J Mol Sci (2022) 23:15591. 10.3390/ijms232415591 36555233 PMC9778880

[27] Thermo Fisher StemPro^®^ osteogenesis differentiation kit (2024). Available from: https://www.thermofisher.com/document-connect/document-connect.html?url=https://assets.thermofisher.com/TFS-Assets%2FLSG%2Fmanuals%2FStemProOsteoDiff.pdf (Accessed March 29, 2024).

[28] Thermo Fisher StemPro^®^ chondrogenesis differentiation kit (2024). Available from: https://www.thermofisher.com/document-connect/document-connect.html?url=https://assets.thermofisher.com/TFS-Assets%2FLSG%2Fmanuals%2Fstempro_chondro_diff_man.pdf (Accessed March 29, 2024).

[29] Thermo Fisher StemPro^®^ adipogenesis differentiation kit (2024). Available from: https://www.thermofisher.com/document-connect/document-connect.html?url=https://assets.thermofisher.com/TFS-Assets%2FLSG%2Fmanuals%2FStemProAdipoDifKit.pdf (Accessed March 29, 2024).

[30] Miltenyi Biotec MSC phenotyping Cocktail kit, anti-human, REAfinity^TM^ (2022). Available from: https://static.miltenyibiotec.com/asset/150655405641/document_1bt1iceg8t1i55jgiiq2gkfq6u?content-disposition=inline (Accessed March 29, 2024).

[31] Mochalova EN Kotov IA Lifanov DA Chakraborti S Nikitin MP. Imaging flow cytometry data analysis using convolutional neural network for quantitative investigation of phagocytosis. Biotechnol Bioeng (2022) 119:626–35. 10.1002/bit.27986 34750809

[32] Bragina VA Khomyakova E Orlov AV Znoyko SL Mochalova EN Paniushkina L Highly sensitive nanomagnetic quantification of extracellular vesicles by immunochromatographic strips: a tool for liquid biopsy. Nanomaterials (Basel) (2022) 12:1579. 10.3390/nano12091579 35564289 PMC9101557

[33] CIF A basic guide to particle characterization (2015). Available from: https://www.cif.iastate.edu/sites/default/files/uploads/Other_Inst/Particle%20Size/Particle%20Characterization%20Guide.pdf (Accessed March 29, 2024).

[34] Banaszynski LA Chen LC Maynard-Smith LA Ooi AG Wandless TJ. A rapid, reversible, and tunable method to regulate protein function in living cells using synthetic small molecules. Cell (2006) 126:995–1004. 10.1016/j.cell.2006.07.025 16959577 PMC3290523

[35] Eren E Wang B Winkler DC Watts NR Steven AC Wingfield PT. Structural characterization of the Myxococcus xanthus encapsulin and ferritin-like cargo system gives insight into its iron storage mechanism. Structure (2022) 30:551–63.e4. 10.1016/j.str.2022.01.008 35150605 PMC8995368

[36] Singh P Pandit S Mokkapati V Garg A Ravikumar V Mijakovic I. Gold nanoparticles in diagnostics and therapeutics for human cancer. Int J Mol Sci (2018) 19:1979. 10.3390/ijms19071979 29986450 PMC6073740

[37] Nikitin MP Zelepukin IV Shipunova VO Sokolov IL Deyev SM Nikitin PI. Enhancement of the blood-circulation time and performance of nanomedicines via the forced clearance of erythrocytes. Nat Biomed Eng (2020) 4:717–31. 10.1038/s41551-020-0581-2 32632229

[38] Ostroverkhov PV Semkina AS Naumenko VA Plotnikova EA Melnikov PA Abakumova TO Synthesis and characterization of bacteriochlorin loaded magnetic nanoparticles (MNP) for personalized MRI guided photosensitizers delivery to tumor. J Colloid Interf Sci (2019) 537:132–41. 10.1016/j.jcis.2018.10.087 30439612

[39] Tregubov A Sokolov I Babenyshev A Nikitin P Cherkasov V Nikitin M. Magnetic hybrid magnetite/metal organic framework nanoparticles: facile preparation, post-synthetic biofunctionalization and tracking *in vivo* with magnetic methods. J Magnetism Magn Mater (2018) 449:590–6. 10.1016/j.jmmm.2017.10.070

[40] Ringaci A Yaremenko A Shevchenko K Zvereva S Nikitin M. Metal-organic frameworks for simultaneous gene and small molecule delivery *in vitro* and *in vivo* . Chem Eng J (2021) 418:129386. 10.1016/j.cej.2021.129386

[41] Lee SW Kim YM Cho CH Kim YT Kim SM Hur SY An open-label, randomized, parallel, phase II trial to evaluate the efficacy and safety of a cremophor-free polymeric micelle formulation of paclitaxel as first-line treatment for ovarian cancer: a Korean gynecologic oncology group study (KGOG-3021). Cancer Res Treat (2018) 50:195–203. 10.4143/crt.2016.376 28324920 PMC5784626

[42] Kovalenko VL Komedchikova EN Sogomonyan AS Tereshina ED Kolesnikova OA Mirkasymov AB Lectin-modified magnetic nano-PLGA for photodynamic therapy *in vivo* . Pharmaceutics (2022) 15:92. 10.3390/pharmaceutics15010092 36678721 PMC9862264

[43] Bae Y Kim GJ Kim H Park SG Jung HS Kang S. Engineering tunable dual functional protein cage nanoparticles using bacterial superglue. Biomacromolecules (2018) 19:2896–904. 10.1021/acs.biomac.8b00457 29847113

[44] Palombarini F Masciarelli S Incocciati A Liccardo F Di Fabio E Iazzetti A Self-assembling ferritin-dendrimer nanoparticles for targeted delivery of nucleic acids to myeloid leukemia cells. J Nanobiotechnology (2021) 19:172. 10.1186/s12951-021-00921-5 34107976 PMC8190868

[45] Orlov AV Nikitin MP Bragina VA Znoyko SL Zaikina MN Ksenevich TI A new real-time method for investigation of affinity properties and binding kinetics of magnetic nanoparticles. J Magnetism Magn Mater (2015) 380:231–5. 10.1016/j.jmmm.2014.10.019

[46] Sanna V Sechi M. Therapeutic potential of targeted nanoparticles and perspective on nanotherapies. ACS Med Chem Lett (2020) 11:1069–73. 10.1021/acsmedchemlett.0c00075 32550978 PMC7294587

[47] Mohammapdour R Ghandehari H. Mechanisms of immune response to inorganic nanoparticles and their degradation products. Adv Drug Deliv Rev (2022) 180:114022. 10.1016/j.addr.2021.114022 34740764 PMC8898339

[48] Stepien G Moros M Perez-Hernandez M Monge M Gutierrez L Fratila RM Effect of surface chemistry and associated protein corona on the long-term biodegradation of iron oxide nanoparticles *in vivo* . ACS Appl Mater Inter (2018) 10:4548–60. 10.1021/acsami.7b18648 29328627

[49] Liu Y Li J. Self-assembling nanoarchitectonics of size-controllable celastrol nanoparticles for efficient cancer chemotherapy with reduced systemic toxicity. J Colloid Interf Sci (2023) 636:216–22. 10.1016/j.jcis.2022.12.162 36634391

[50] Putri RM Allende-Ballestero C Luque D Klem R Rousou KA Liu A Structural characterization of native and modified encapsulins as nanoplatforms for *in vitro* catalysis and cellular uptake. ACS Nano (2017) 11:12796–804. 10.1021/acsnano.7b07669 29166561 PMC6150732

[51] Zhang D Lee HF Pettit SC Zaro JL Huang N Shen WC. Characterization of transferrin receptor-mediated endocytosis and cellular iron delivery of recombinant human serum transferrin from rice (Oryza sativa L). BMC Biotechnol (2012) 12:92. 10.1186/1472-6750-12-92 23194296 PMC3521190

[52] Gu Z Wang H Xia J Yang Y Jin Z Xu H Decreased ferroportin promotes myeloma cell growth and osteoclast differentiation. Cancer Res (2015) 75:2211–21. 10.1158/0008-5472.can-14-3804 25855377 PMC4946247

[53] Ohkuma M Haraguchi N Ishii H Mimori K Tanaka F Kim HM Absence of CD71 transferrin receptor characterizes human gastric adenosquamous carcinoma stem cells. Ann Surg Oncol (2012) 19:1357–64. 10.1245/s10434-011-1739-7 21523522

[54] Singh M Mugler K Hailoo DW Burke S Nemesure B Torkko K Differential expression of transferrin receptor (TfR) in a spectrum of normal to malignant breast tissues: implications for *in situ* and invasive carcinoma. Appl Immunohistochem Mol Morphol (2011) 19:417–23. 10.1097/pai.0b013e318209716e 21297444

[55] Czarnywojtek A Borowska M Dyrka K Van Gool S Sawicka-Gutaj N Moskal J Glioblastoma multiforme: the latest diagnostics and treatment techniques. Pharmacology (2023) 108:423–31. 10.1159/000531319 37459849

[56] Nikitin MP. Non-complementary strand commutation as a fundamental alternative for information processing by DNA and gene regulation. Nat Chem (2023) 15:70–82. 10.1038/s41557-022-01111-y 36604607

[57] Vasileva AV Gladkova MG Ashniev GA Osintseva ED Orlov AV Kravchuk EV Super-enhancers and their parts: from prediction efforts to pathognomonic status. Int J Mol Sci (2024) 25:3103. 10.3390/ijms25063103 38542080 PMC10969950

[58] Moeinabadi-Bidgoli K Mazloomnejad R Beheshti Maal A Asadzadeh Aghdaei H Kazem Arki M Hossein-Khannazer N Genetic modification and preconditioning strategies to enhance functionality of mesenchymal stromal cells: a clinical perspective. Expert Opin Biol Ther (2023) 23:461–78. 10.1080/14712598.2023.2205017 37073114

[59] Gabashvili AN Efremova MV Vodopyanov SS Chmelyuk NS Oda VV Sarkisova VA New approach to non-invasive tumor model monitoring via self-assemble iron containing protein nanocompartments. Nanomaterials (2022) 12:1657. 10.3390/nano12101657 35630878 PMC9145190

[60] Fedotov K Efremova M Gabashvili A Semkina A Sigmund F Sarkisova V Towards multiscale tracking of stem cells with genetically encoded encapsulin nanocompartments. FEBS OPEN BIO (2021) 273–3. WILEY 111 RIVER ST, HOBOKEN 07030-5774, NJ USA.

[61] Rennie C Sives C Boyton I Diaz D Gorrie C Vittorio O *In vivo* behavior of systemically administered encapsulin protein nanocages and implications for their use in targeted drug delivery. Adv Ther (2024) 7:2300360. 10.1002/adtp.202300360

