# Supplementary material for: Internalization of transferrin-tagged Myxococcus xanthus encapsulins into mesenchymal stem cells
Source: Exp Biol Med (Maywood). 2024 May 7;249:10055. doi: 10.3389/ebm.2024.10055 (PMC11106444; doi:10.3389/ebm.2024.10055)
Supplement: Supplementary file 1 [file DataSheet1.docx]

Supplementary Materials: Transferrin-tagged *Myxococcus xanthus* encapsulins internalization into mesenchymal stem cells via receptor-mediated endocytosis

Anna N. Gabashvili, Natalya A. Alexandrushkina, Elizaveta N. Mochalova, Daria V. Goliusova, Ekaterina N. Sapozhnikova, Pavel I. Makarevich, and Petr I. Nikitin


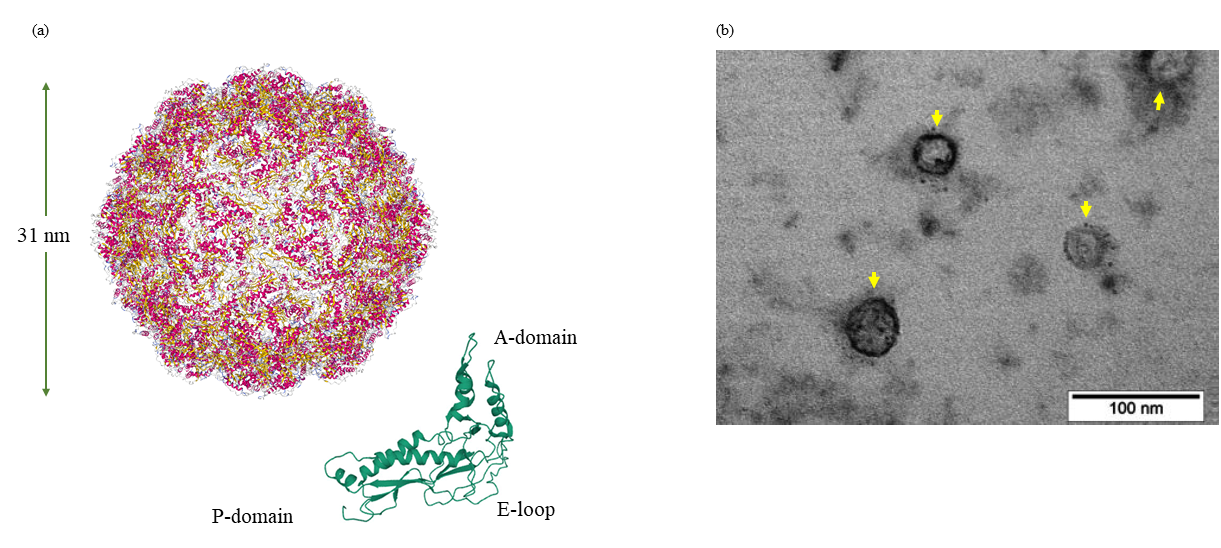


(b)

(a)

Figure S1. (a) *Myxococcus xanthus* encapsulin shell EncA protein with T=3 symmetry (PDB: 7S20). The structure of an encapsulin shell protomer protein has three conserved domains: a peripheral domain (P), an axial domain (A), and an elongated loop (E). (b) Bright-field TEM image of Uranyless - stained Mx encapsulins protein shells (yellow arrows), scale bar 100 nm (10.3390/ijms232415591). T (the triangulation number) - the quotient of dividing the number of subunits in a shell by 60.


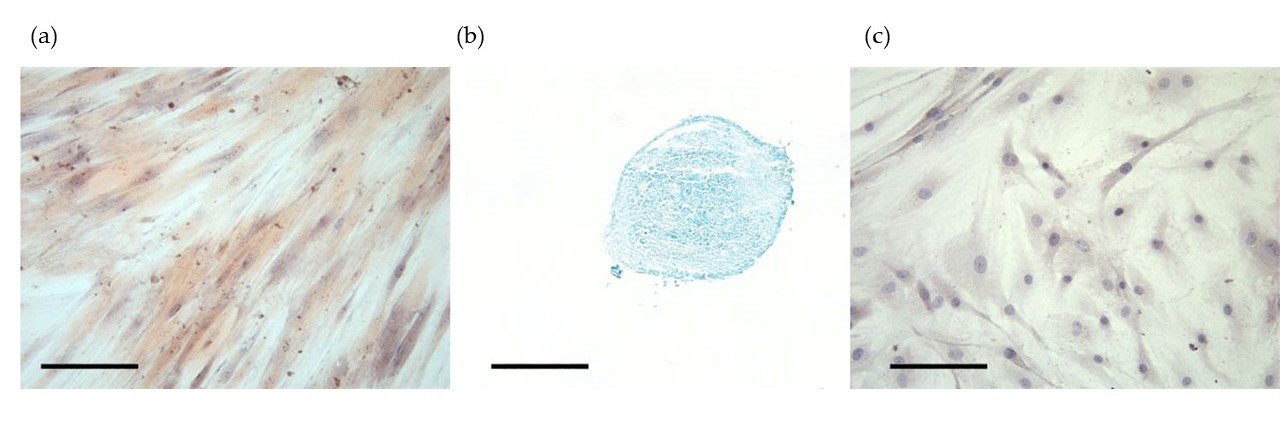


Figure S2. Control MSCs (no differentiation medium added) stained with: (a) Alizarin Red, (b) Alcian Blue, and (c) Oil Red. Scale bars are 50 μm.


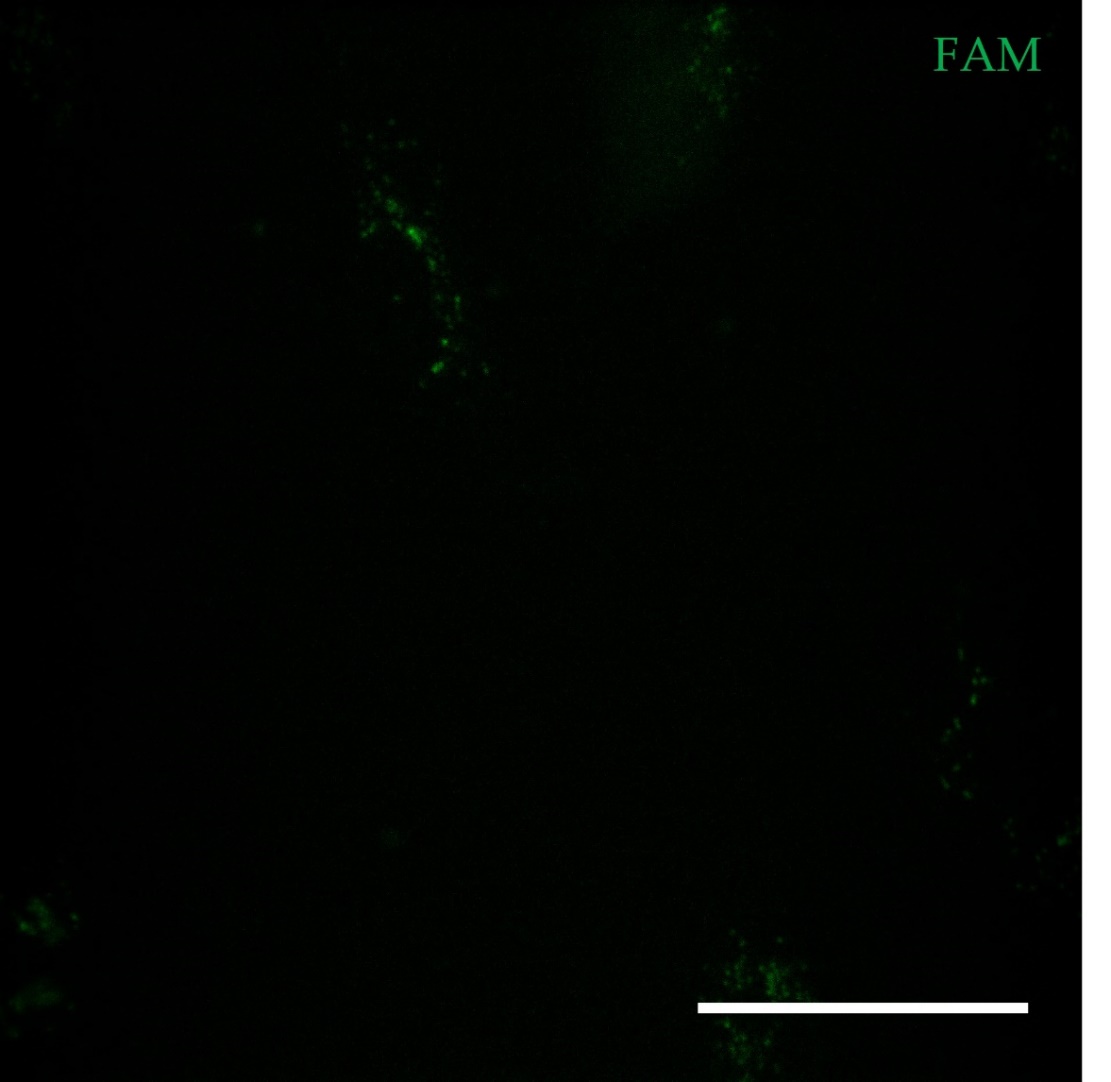


Figure S3. Uptake of Tf-FAM in human fibroblasts after 90 min of incubation. Green fluorescent signal – FAM. Laser scanning confocal microscopy, Nikon Eclipse Ti2, scale bar 50 μm.


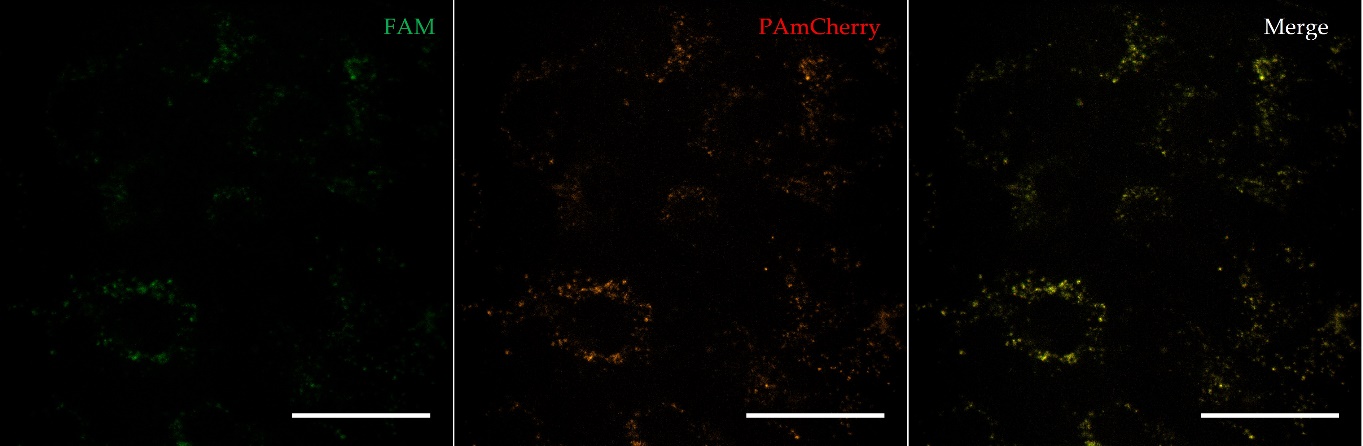


Figure S4. Uptake and intracellular distribution of Mx-Tf-FAM in human fibroblasts after 90 min of incubation. Green fluorescent signal – FAM, red fluorescent signal – PAmCherry. Nikon Eclipse Ti2, scale bars are 50 μm.

(b)

(a)


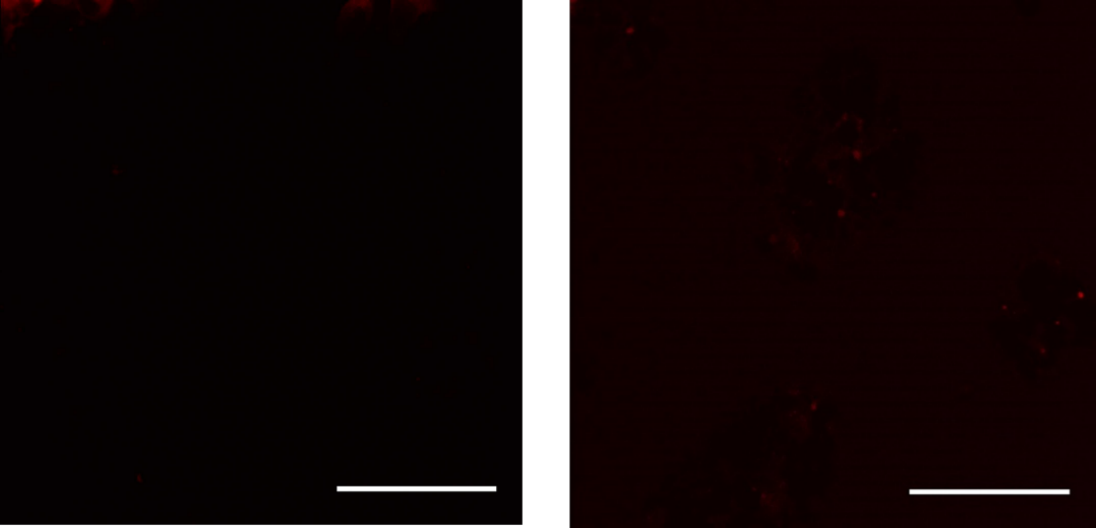


Figure S5. Uptake of isolated non-vector Mx encapsulins in human MSCs (a) and fibroblasts (b) after 120 min of incubation. Nikon Eclipse Ti2, scale bars are 50 μm.
